# Impaired Neonatal Immunity and Infection Resistance Following Fetal Growth Restriction in Preterm Pigs

**DOI:** 10.3389/fimmu.2020.01808

**Published:** 2020-08-13

**Authors:** Ole Bæk, Shuqiang Ren, Anders Brunse, Per Torp Sangild, Duc Ninh Nguyen

**Affiliations:** Section for Comparative Pediatrics and Nutrition, University of Copenhagen, Copenhagen, Denmark

**Keywords:** preterm, infant, small for gestation age, fetal growth restriction, immunity, neonatal sepsis

## Abstract

**Background:** Infants born preterm or small for gestational age (SGA, due to fetal growth restriction) both show an increased risk of neonatal infection. However, it remains unclear how the co-occurrence of preterm birth and SGA may affect neonatal immunity and infection risk. We hypothesized that fetal growth restricted (FGR) preterm newborns possess impaired immune competence and increased susceptibility to systemic infection and sepsis, relative to corresponding normal birth weight (NBW) newborns.

**Methods:** Using preterm pigs as a model for preterm infants, gene expression in lipopolysaccharide (LPS) stimulated cord blood was compared between NBW and FGR (lowest 25% birth weight percentile) preterm pigs. Next, clinical responses to a systemic *Staphylococcus epidermidis* (SE) challenge were investigated in newborn FGR and NBW preterm pigs. Finally, occurrence of spontaneous infections were investigated in 9 d-old FGR and NBW preterm pigs, with or without neonatal antibiotics treatment.

**Results:** At birth, preterm FGR piglets showed diminished *ex vivo* cord blood responses to LPS for genes related to both innate and adaptive immunity, and also more severe septic responses following SE infection (e.g., higher blood lactate, decreased blood pH, neutrophil and platelet counts, relative to NBW pigs). After 9 d, FGR pigs had higher incidence and severity of spontaneous infections (e.g., higher bacterial densities in the bone marrow), increased regulatory T cell numbers, reduced neutrophil phagocytosis capacity, and impaired *ex vivo* blood gene responses to LPS, especially when receiving neonatal antibiotics.

**Conclusion:** FGR at preterm birth is associated with poor immune competence, impaired infection resistance, and greater sepsis susceptibility in the immediate postnatal period. Our results may explain the increased morbidity and mortality of SGA preterm infants and highlight the need for clinical vigilance for this highly sensitive subgroup of preterm neonates.

## Background

Preterm infants (born before 37 weeks of gestation) are at a higher risk of life threatening infections than their term born counterparts, risk increasing with lower gestational age (1–3). The causes of this are multifaceted, including an immature immune system, comorbidities related to prematurity, and iatrogenic interventions during hospital admission (3, 4). In addition, intrauterine complications that could lead to premature birth, may also affect nutrient supply to the fetus. If fetal growth is severely affected, infants could be born small for gestational age (SGA), defined as a birth weight in the lower 10th percentile of the expected weight for gestational age. Infants born SGA, whether preterm or not, show a higher postnatal mortality than infants of adequate birth weight (5). They also display lower blood neutrophil counts at birth and less responsive leucocytes to *ex vivo* infectious challenges (6, 7). Some studies even suggested that this impaired immune status may persist for years after birth with SGA (8, 9). From animal studies, low birth weight following term birth has been correlated with negative long term effects on growth as well as systemic and gut immune functions (10–13). The possible effects of being both premature and SGA on immune development and infection risks during the first few days of life, remain elusive. Few observational studies indicate an association between SGA and increased mortality and sepsis in preterm infants (3, 14–16) and cord blood from SGA preterm infants may be less responsive to *ex vivo* lipopolysaccharide (LPS) challenge (17). Regardless, the separate effects of prematurity and SGA remain unclear because both overlapping and independent factors may predispose to preterm delivery and growth restriction at birth (e.g., maternal infection/inflammation, poor placental function, reduced blood supply/oxygenation, or genetic factors).

Systemic infection in preterm infants is difficult to diagnose due to the lack of precise biomarkers and poor sensitivity of blood culture assays using small blood volumes (18–20). Therefore, empirical antibiotics are used for a majority of preterm infants in the days after birth despite that only a fraction these infants may indeed be infected (21, 22). Such antibiotic treatments in the neonatal period may affect gut bacterial colonization and immune development early in life, although the evidence from infants are limited. In preterm pigs, neutrophil status is affected by prophylactic oral antibiotics (23). In term pigs, short term neonatal antibiotics altered immune response several weeks after exposure, even though the changes to gut microbiota disappeared after 1 week (24). In term infants, early life antibiotic use is associated with later development of asthma and eczema (25, 26), but such studies are of limited value for the special condition of preterm birth. More information is required on the possible interacting effects of antibiotics treatment and growth restriction on immune development in the early life of preterm neonates.

Preterm pigs are acknowledged as a clinically relevant model for preterm infants as they show many complications similar to preterm infants, including impaired immunity, immature organ systems, and increased susceptibility to sepsis and necrotizing enterocolitis (27, 28). Planned delivery by preterm cesarean section on pregnant sows makes it possible to study factors related to reduced gestational age at birth (e.g., developmental immaturity), independent from the pathological factors that may predispose to and affect outcomes in preterm infants, besides reduced fetal age. Using the preterm pig model, we hypothesized that fetal growth restriction (FGR) would further impair immune competence and increase the risk of systemic infection and sepsis in preterm pigs, relative to preterm pigs with a normal body weight (NBW) for their gestational age. FGR was defined as the lowest 25% birth weight percentile to include both moderately and extremely growth restricted neonates in evaluating immune status, spontaneous infections and responses to infection challenges, with or without antibiotic treatment just after preterm birth. Different from our previous longer term study on systemic immune status in FGR and NBW preterm pigs with optimal feeding to avoid clinical complications (10), we now focused on the *in vitro* and *in vivo* responses to systemic infection in the first week after preterm birth when both preterm pigs and infants are highly sensitive to infections.

## Methods

All animal experiments were approved by the Danish National Committee on Animal Experimentation (2014-15-0201-00418). We performed three experiments to determine the immune competence, sepsis outcomes following infection challenges and spontaneous infection in FGR preterm pigs. All pigs were of the same race (Duroc x Yorkshire x Danish Landrace) and delivered by cesarean section at the same gestational age (106 days, ~90% gestation), probably reflecting an immune system development of human preterm infants born at 24–28 weeks of gestation, as described previously (27). Within each litter across these experiments, FGR animals were defined as those in the lower 25th percentile for birth weight, while remaining animals were designated as normal birth weight (NBW).

### Experiment 1

In order to explore immune competence at birth, cord blood was collected from 81 preterm pigs (21 FGR, 60 NBW) across 4 litters from the umbilical cord at delivery. Fresh blood was then immediately used for *ex vivo* stimulation assay as described later.

### Experiment 2

Fifty-seven preterm pigs across 5 litters were delivered, immediately resuscitated in heated and oxygenated incubators (2 L/min) and manual ventilation was performed if deemed necessary. While still affected by anesthesia, each pig was fitted with an umbilical arterial catheter (4F, Portex, UK) for arterial blood sampling and administration of parenteral nutrition. Shortly after birth, 38 animals were infused with live *Staphylococcus epidermidis* (SE) bacteria (1^*^10^8^ − 5^*^10^9^ colony forming units/kg) via the umbilical catheter, as previously described (29). The pigs were stratified by the predefined FGR criteria, resulting in two groups SE-FGR (*n* = 9) and SE-NBW (*n* = 29). The remaining pigs received the same volume of control saline, and were not divided by birth weight due to the low number (CON, *n* = 19). Following administration of SE or saline, pigs were closely monitored for the next 24 h for signs of sepsis, pain, and circulatory collapse. If severe symptoms appeared, animals were euthanized ahead of schedule according to predefined, humane endpoints. During the experiment, animals were kept on total parenteral nutrition (Kabiven, Fresenius-Kabi, Swenden, 6 mL/kg/h). Blood samples were drawn from the umbilical catheter 6, 12, and 24 h after inoculation and used for hematology and arterial blood gas analysis, as described later. After 24 h, all pigs were euthanized by intracardial injection of pentobarbital.

### Experiment 3

Preterm pigs (*n* = 127) across 7 litters were delivered by cesarean section, resuscitated and fitted with umbilical catheters as in *Exp 2*. In addition, the pigs were fitted with an orogastric feeding tube (6F, Portex, UK) for administration of enteral nutrition and antibiotics. A proportion of animals (*n* = 30) received oral antibiotics within the first 4 days of life. All pigs were reared until postnatal day 9. The animals were divided by birth weight (NBW or FGR) and antibiotic use (CON or AB), resulting in four groups: NBW-CON (*n* = 78), FGR-CON (*n* = 19), NBW-AB (*n* = 22), and FGR-AB (*n* = 8).

The pigs were weighed daily and clinically assessed twice a day, for signs of illness or pain. If necessary, a pain relief drug was given as an intramuscular injection of meloxicam or butorphanol (Metacam, Denmark or Torbugesic, Finland). According to predefined humane endpoints, animals were euthanized ahead of schedule if they exhibited treatment resistant pain or severe morbidities. The pigs were fed, via the orogastric catheter, increasing amounts of infant formula (16–112 mL/kg/day). Pigs received different bovine milk based diets, that were prepared daily, as described previously (30). The enteral nutrition was supported by decreasing amount of parenteral nutrition (same formulation as in *Exp 2*, from 6 mL/kg/h at birth to 2 mL/kg/h on days 6–9). For NBW-AB and FGR-AB pigs, a combination of amoxicillin with clavulanic acid (50/25 mg/kg, Bioclavid, Sandoz GmbH, Austria) and neomycin (50 mg/kg, Neomay, ScanVet, Denmark) was given through the orogastric tube twice daily for the first 4 days. Blood samples for the evaluation of hematology, neutrophil phagocytosis, T cell profiling, and gene expression analysis were drawn from the umbilical catheter on day 5, 7, and 9, after which all pigs were euthanized by intercardial injection of pentobarbital. After euthanasia the left hind leg was severed, the femur head dissected and immersed in 70% ethanol for 10 min, and a bone marrow biopsy was collected in a sterile manner, for bacterial enumeration.

### Immune Cell Characterization

Hematology and immune cell counts on all blood samples from *Exp 2* and *3* were performed on an Advia 2120 hematology system (Siemens Healthcare Diagnostics, USA). In *Exp 2* arterial blood gas analysis was performed using a GEM Premier 3000 (Instrumentation Laboratory, USA).

In *Exp 3*, T cell characterization was performed as previously described (31). Briefly, blood leucocytes were stained with fluorescent antibodies against porcine CD3, CD4, CD8, and FOXP3. Leucocytes were then analyzed using a BD Accuri C6 flow cytometer (BD Biosciences, USA). T cell subsets were defined as follows: T cells (CD3^+^ lymphocytes), CD4 positive T cells (CD3^+^CD4^+^CD8^−^ lymphocytes), CD8 positive T cells (CD3^+^CD4^−^CD8^+^ lymphocytes) and regulatory T cells (CD3^+^CD4^+^FOXP3^+^ lymphocytes).

### Whole Blood Stimulation and Neutrophil Phagocytosis Assays

*Ex vivo* whole blood stimulation and leucocyte gene expression was performed in *Exp 1* and in a subgroup of *Exp 3* (NBW-CON, *n* = 25; FGR-CON *n* = 5; NBW-AB, *n* = 19; FGR-AB, *n* = 7). Briefly, fresh whole blood was incubated at 37°C for 5 h with or without 1 μg/ml LPS added. After stimulation, whole blood was stabilized with a mixture of lysis/binding solution concentrate and isopropanol (MagMax 96 blood RNA isolation kit, Thermofisher, Roskilde, Denmark), and stored at −80°C until RNA extraction. RNA was extracted and prepared for quantitative polymerase chain reaction (qPCR) as described elsewhere (10). Using a LightCycler 480 system (Roche, Switzerland) and a commercial qPCR kit (QuantiTect SYBR Green PCR Kit, Qiagen, Netherlands), gene expressions were determined for a panel of 23 genes related to innate and adaptive immunity, as well as cellular metabolism. The genes and corresponding primers are shown in Table 1, primers were designed using the *Genes* database and Primer-BLAST software (both National Center for Biotechnology Information, USA). *HPRT1* was used as a housekeeping gene. If a gene in a sample could not be detected by qPCR the sample was rerun, if no expression could be obtained the gene was censored from analysis. If *HPRT1* could not be determined, the entire sample was censored from analysis. Differences in expression of specific genes were calculated relative to the expression of the housekeeping gene, done separately in LPS and non-LPS stimulated samples.

**Table 1:** List of genes and primers used in gene expression analysis.

**Protein**	**Gene**	**Forward sequence (5**′**-3**′**)**	**Reverse sequence (5**′**–3**′**)**	**Amplicon length**
CXC chemokine ligand 9	*CXCL9*	GAAAAGCAGTGTTGCCTTGCT	TGATGCAGGAACAACGTCCAT	98
CXC chemokine ligand 10	*CXCL10*	ATCATCCCGAGCTGTTGAGC	CCAGGACTTGGCACATTCAC	94
GATA binding protein 3	*GATA3*	ACCCCTTATTAAGCCCAAGC	TCCAGAGAGTCGTCGTTGTG	92
Hypoxia inducible factor 1 alpha	*HIF1A*	TGTGTTATCTGTCGCTTTGAGTC	TTTCGCTTTCTCTGAGCATTC	96
Hexokinase 1	*HK1*	TTTCCCTTGTCGGCAATCCA	CCTCCACTCCGCTTGCTTTA	80
Hypoxanthine phosphoribosyltransferase 1	*HPRT1*	TATGGACAGGACTGAACGGC	ACACAGAGGGCTACGATGTG	75
Interferon gamma	*IFNG*	AGCTTTGCGTGACTTTGTGT	ATGCTCCTTTGAATGGCCTG	247
Interleukin 2	*IL2*	AAGCTCTGGAGGGAGTGCTA	CAACAGCAGTTACTGTCTCATCA	159
Interleukin 4	*IL4*	GTACCAGCAACTTCGTCCAC	CCTTCTCCGTCGTGTTCTCT	150
Interleukin 6	*IL6*	TGCCACCTCAGACAAAATGC	AGGTTCAGGTTGTTTTCTGCC	159
Interleukin 10	*IL10*	GTCCGACTCAACGAAGAAGG	GCCAGGAAGATCAGGCAATA	73
Interleukin 12	*IL12*	TCCTGGGAAAGTCCTGTCGT	GGTGAGGTCGCTAGTTTGGA	81
Interleukin 17	*IL17*	GCACACGGGCTGCATCAACG	TGCAACCAACAGTGACCCGCA	149
Myeloperoxidase	*MPO*	CCCGAGTTGCTTTCCTCACT	AAGAAGGGGATGCAGTCACG	127
Pyruvate dehydrogenase α1	*PDHA1*	GTCAGGAAGCTTGTTGCGTG	GGTAAAGCCATGAGCTCGGT	86
Pyruvate kinase	*PKM*	GCCCTGGACACTAAAGGACC	CAGCCACAGGACATTCTCGT	147
Peroxisome proliferator activated receptor alpha	*PPARA*	CCGAGACCGCAGATCTCAAG	GACGAAAGGCGGGTTATTGC	128
RAR-related orphan receptor alpha	*RORrt*	CAGCGCTCCAACATCTTCTC	GACCAGCACCACTTCCATTG	207
S100 Calcium Binding Protein A9	*S100A9*	GCCAAACTTTCTCAAGAAGCA	AGTGTCCAGGTCTTCCAGGAT	70
T-Box transcription factor	*TBET*	CTGAGAGTCGCGCTCAACAA	ACCCGGCCACAGTAAATGAC	121
Transforming growth factor beta 1	*TGFB1*	GCAAGGTCCTGGCTCTGTA	TAGTACACGATGGGCAGTGG	97
Toll-like receptor 2	*TLR2*	CGTGTGCTATGACGCTTTCG	GTACTTGCACCACTCGCTCT	232
Toll-like receptor 4	*TLR4*	TGGTGTCCCAGCACTTCATA	CAACTTCTGCAGGACGATGA	116
Tumor necrosis factor alpha	*TNFA*	ATTCAGGGATGTGTGGCCTG	CCAGATGTCCCAGGTTGCAT	120

In the same subgroup of *Exp 3*, neutrophil phagocytic function was assessed using commercial fluorescently labeled *E. coli* kit (pHrodo, ThermoFisher, Roskilde, Denmark), as described previously (32). In short, whole blood was incubated with the fluorescent bacteria, and thereafter analyzed on the above mentioned flow cytometer. Neutrophils were identified and phagocytic rate was defined as the fraction of neutrophils with internalized bacteria and phagocytic capacity, as the median fluorescent index of neutrophils with internalized bacteria.

### Microbiology

In *Exp 3* we assessed spontaneous bacterial accumulation in the bone marrow. Bone marrow homogenate was serially diluted, plated out on blood agar plates and incubated for 24 h at 37°C. Colonies were counted and bacterial density was assessed as colony forming units per milliliter of bone marrow homogenate. Later, bacteria were identified at species level by Matrix Assisted Laser Desorption/Ionization time of flight mass spectroscopy, as previously described (23).

### Statistics

Stata 14.2 (StataCorp, Texas, USA) was used for all calculations. For all comparisons, we used a linear mixed effect model, with relevant intervention as the fixed factor and litter as the random factor. If a variable could not conform to normal distribution after logistic transformation it was assessed by a non-parameteric, Kruskal Wallis' test. In *Exp 1* differences between FGR and NBW animals in gene expression were assessed separately with or without LPS stimulation. Afterwards the effect of LPS stimulations was determined by a similar model using LPS as the fixed factor and the individual pigs as the random factor. In *Exp 2*, within the animals inoculated with SE we first tested the effect of FGR, separately for each post inoculation time point. To elucidate the effect of SE inoculation we compared all SE infused animals (both FGR and NBW) to all saline infused animals (CON). One litter (*n* = 24) was removed from this comparison as it only included SE infused pigs and no saline infused control group. In *Exp 3* we first tested the effects of FGR on bone marrow infection and blood endpoints separately in antibiotic and non-antibiotic treated preterm pigs at each postnatal time point. The gene expression analysis on day 5 and 9 was assessed in the same manner as in *Exp 1*.

## Results

### Impaired Immune Competence in FGR Pigs at Birth Assessed by Cord Blood LPS Stimulation

**Figure 1:**
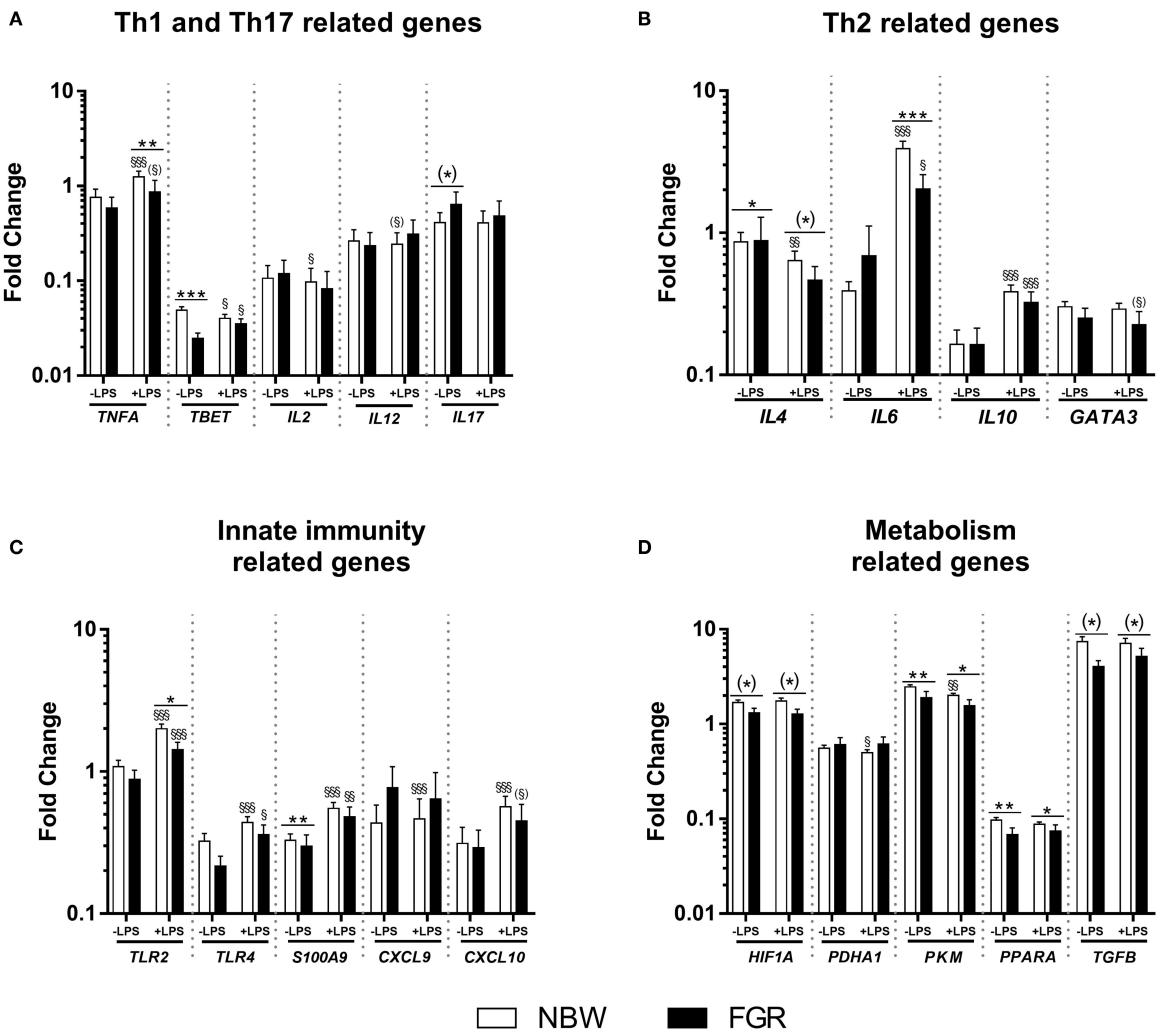
Cord blood leucocyte gene expressions in normal birth weight (NBW, *n* = 60) and fetal growth restricted (FGR, *n* = 21) preterm pigs. Expression of **(A)** Th1 and Th17–, **(B)** Th2–, **(C)** innate immunity-, and **(D)** cellular metabolism-related genes. Data are shown as fold changes in relation to housekeeping gene, all presented on a logarithmic scale, before and after stimulation with lipopolysaccharide (LPS). Data are presented as means with corresponding standard error. *: Difference between NBW and FGR; (*) *P* < 0.1, *P < 0.05, **P < 0.01, ***P < 0.001. ^§^: Effect of LPS stimulation; (^§^)*P* < 0.1, ^§^*P* < 0.05, ^§§^*P* < 0.01, ^§§§^*P* < 0.001.

In *Exp 1*, the mean birth weight was lower in the FGR group compared to the NBW (723 ± 30 vs. 1,119 ± 19 g, *P* < 0.001). Before LPS stimulation in cord blood, FGR preterm pigs showed diminished expression of *TBET* and *SA100A9* as well slightly higher expression of *IL4* (*P* < 0.01, 0.001, and 0.05, respectively, Figures 1A–C). After LPS stimulation, the FGR group showed lower expression of *TNFA, IL6* and *TLR2* (*P* < 0.01, 0.001, and 0.05, respectively, Figures 1A–C), with tendencies toward lower expression of *IL4* (*P* = 0.07, Figure 1B), relative to NBW pigs. In the FGR group, LPS stimulation increased expression of *TBET, IL6, IL10, TLR2, TLR4*, and *S100A9* (all *P* < 0.05, Figures 1A–C), with tendencies toward higher expression of *TNFA* and *CXCL10* (*P* = 0.09 and 0.06, respectively, Figures 1A,C) and lower expression of *GATA3* (*P* = 0.06, Figure 1B). Within the NBW group, LPS stimulation increased expression of *TNFA, IL6, IL10, TLR2, TLR4, S100A9, CXCL9*, and *CXCL10* (all *P* < 0.01, Figures 1A–C) and diminished expression of *TBET, IL2* and *IL4* (*P* < 0.05, 0.05, and 0.01, respectively, Figures 1A,B). There was also a tendency toward less expression of *IL12* (*P* = 0.07, Figure 1A).

For genes related to cellular metabolism, FGR pigs showed lower expression of *PPARA* and *PKM* before LPS stimulation (all *P* < 0.01, Figure 1D), with a tendency toward lower expression of *HIF1A* and *TGFB* (*P* = 0.08 and 0.07, respectively, Figure 1D) than NBW pigs. After LPS stimulation, the FGR group still showed lower expression of *PPARA* and *PKM* (all *P* < 0.05, Figure 1D) and a tendency toward lower expression of *HIF1A* and *TGFB* (both *P* = 0.07, Figure 1D). Within the NBW group, LPS stimulation lead to lower expression of *PADHA1 and PKM* (all *P* < 0.05, Figure 1D). Within the FGR group, LPS stimulation did not affect the expression of any metabolism related gene.

### Severe Sepsis Outcomes in Newborn Preterm FGR Pigs Following SE Infection

**Figure 2:**
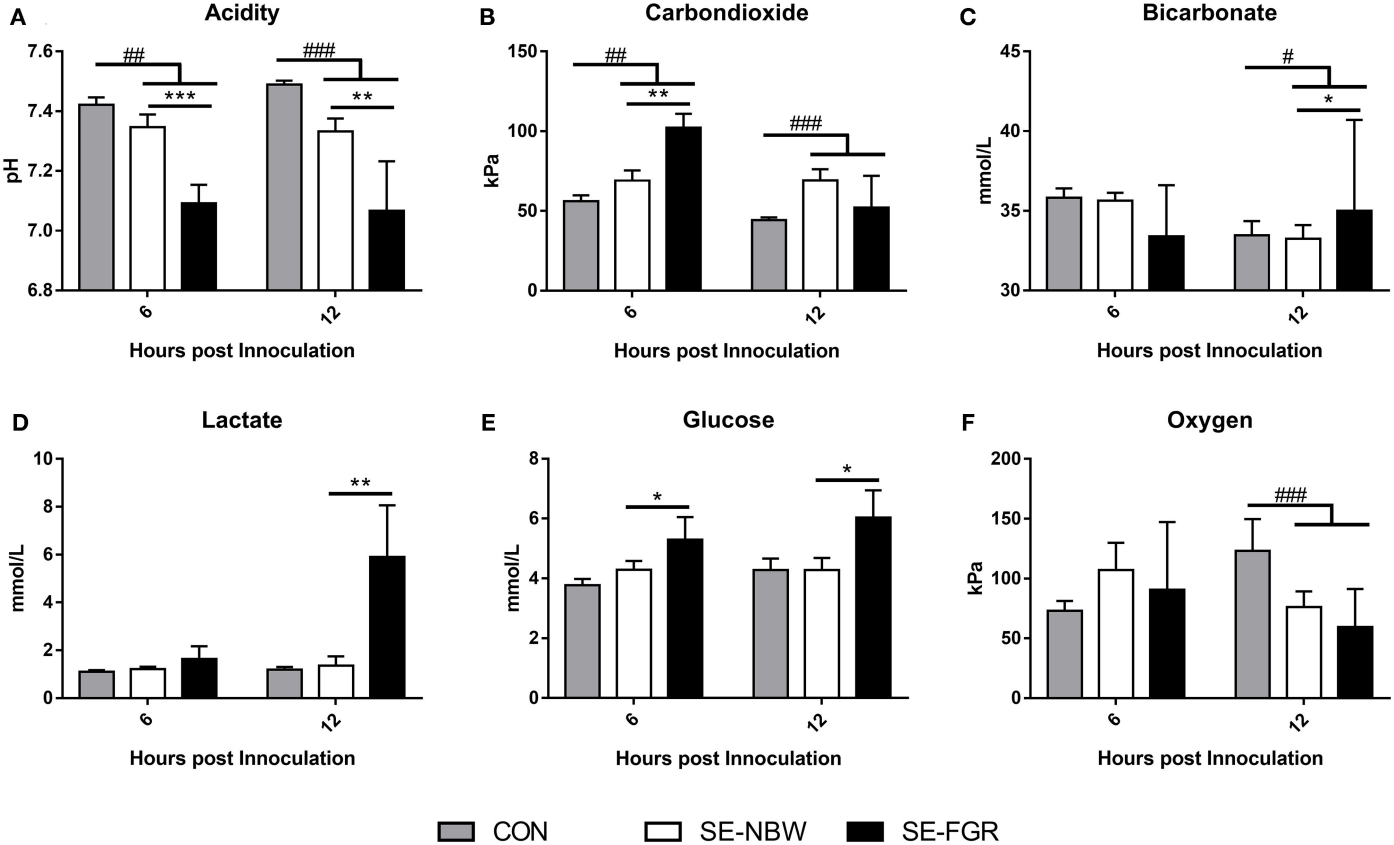
Blood gas parameters in normal birth weight and fetal growth restricted preterm pigs inoculated with live *Staphylococcus epidermidis* (SE-NBW, *n* = 29 and SE-FGR, *n* = 9, respectively), as well as preterm pigs inoculated with control saline (CON, *n* = 19). **(A)** Blood pH. **(B)** Blood carbondioxide pressure. **(C)** Blood levels of bicarbonate. **(D)** Blood levels of lactate. **(E)** Blood glucose levels. **(F)** Blood oxygen pressure Data are presented as means with corresponding standard error, 6 and 12 h after inoculation. ^#^: Difference between all SE inoculated preterm pigs and CON; ^#^*P* < 0.05, ^##^*P* < 0.01, ^###^*P* < 0.001. *: Difference between SE-NBW and SE-FGR; *P < 0.05, **P < 0.01, ***P < 0.001.

In *Exp 2*, the mean birth weight was lower in SE-FGR than SE-NBW (701 ± 38 vs. 993 ± 34 g, *P* < 0.001). The overall birth weight did not differ between SE inoculated animals and CON (data not shown). Following SE infusion, SE inoculated pigs showed septic responses with lower blood pH and higher blood carbon dioxide pressure at 6 and 12 h (all *P* < 0.01, Figures 2A,B), and higher bicarbonate levels and lower oxygen pressure at 12 h after inoculation (*P* < 0.05 and 0.001, respectively, Figures 2C,F). Relative to SE-NBW pigs, SE-FGR preterm pigs had lower blood pH 6 h after inoculation with corresponding higher carbon dioxide pressure (all *P* < 0.01, Figures 2A,B). After 12 h, pH was still lower in SE-FGR with higher bicarbonate and lactate levels (Figures 2A,C,E). Blood glucose levels were consistently higher in SE-FGR than SE-NBW pigs at both 6 and 12 h (*P* < 0.05, Figure 2F).

**Figure 3:**
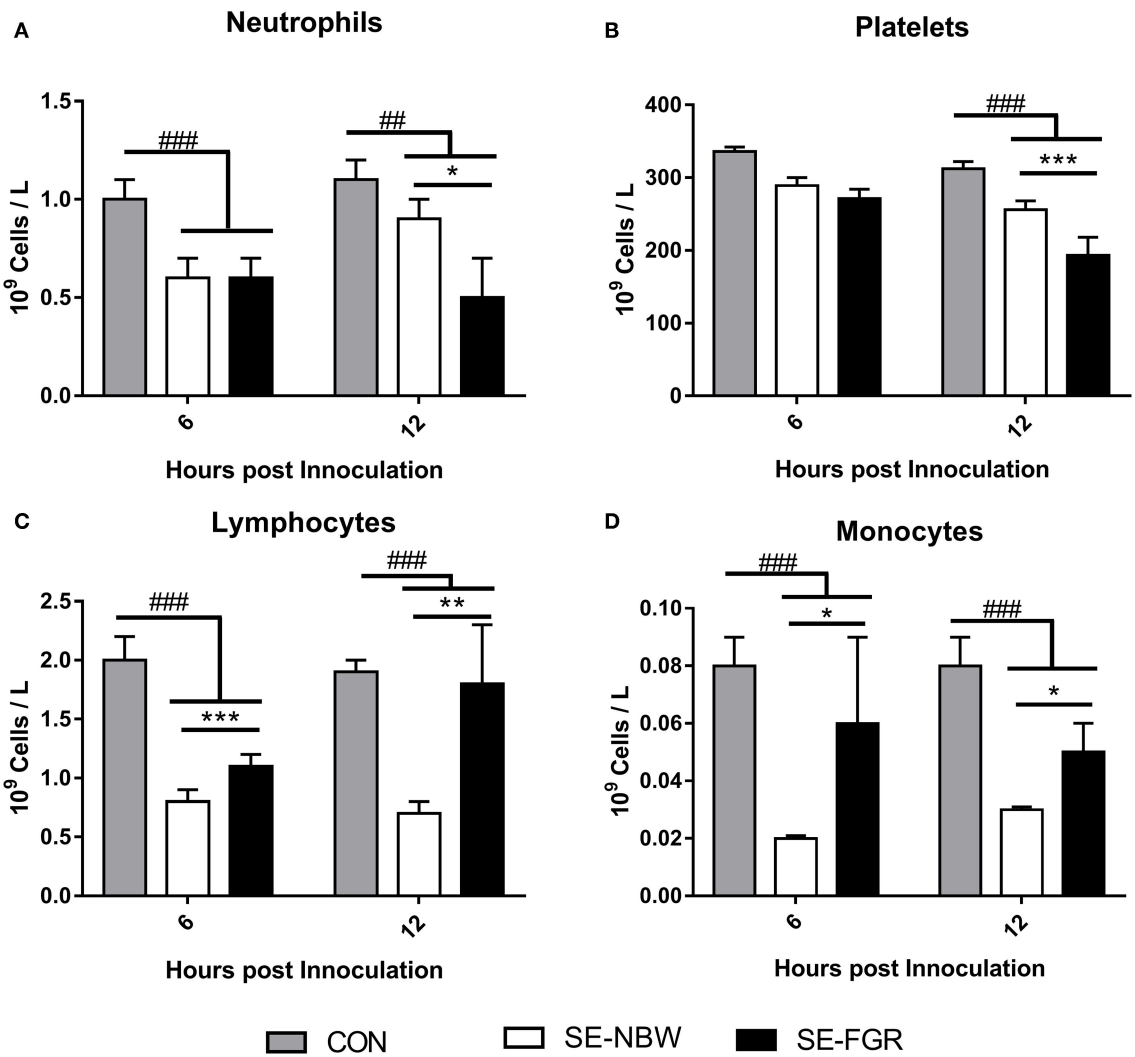
Hematological parameters in normal birth weight and fetal growth restricted preterm pigs inoculated with live *Staphylococcus epidermidis* (SE-NBW, *n* = 29 and SE-FGR, *n* = 9, respectively), as well as preterm pigs inoculated with control saline (CON, *n* = 19). **(A–D)** Blood neutrophil, lymphocyte, platelet, and monocyte counts. Data are presented as means with corresponding standard error, 6 and 12 h after inoculation. ^#^: Difference between all SE inoculated preterm pigs and CON. ^*##*^*P* < 0.01, ^*###*^*P* < 0.001. *: Difference between SE-NBW and SE-FGR; *P < 0.05, **P < 0.01, ***P < 0.001.

For the cellular immune parameters, relative to controls, SE infected pigs experienced depletion of blood neutrophils, lymphocytes, and monocytes at 6 and 12 h (all *P* < 0.001, Figures 3A,B,D) with lower platelet counts only at 12 h (*P* < 0.001, Figure 3B). Relative to SE-NBW pigs, SE-FGR pigs showed blood lower neutrophil and platelet counts after 12 h (*P* < 0.05 and 0.001, respectively, Figures 3A,B). Blood lymphocyte and monocyte counts were higher in SE-FGR vs. SE-NBW pigs after 6 and 12 h (all *P* < 0.05, respectively, Figure 3C). The full panel of hematological parameters at 6, 12, and 24 h after inoculation was shown in Supplementary Table 1.

### Impaired Immune Competence and Increased Nosocomial Infection in Neonatal Preterm FGR Pigs

**Figure 4:**
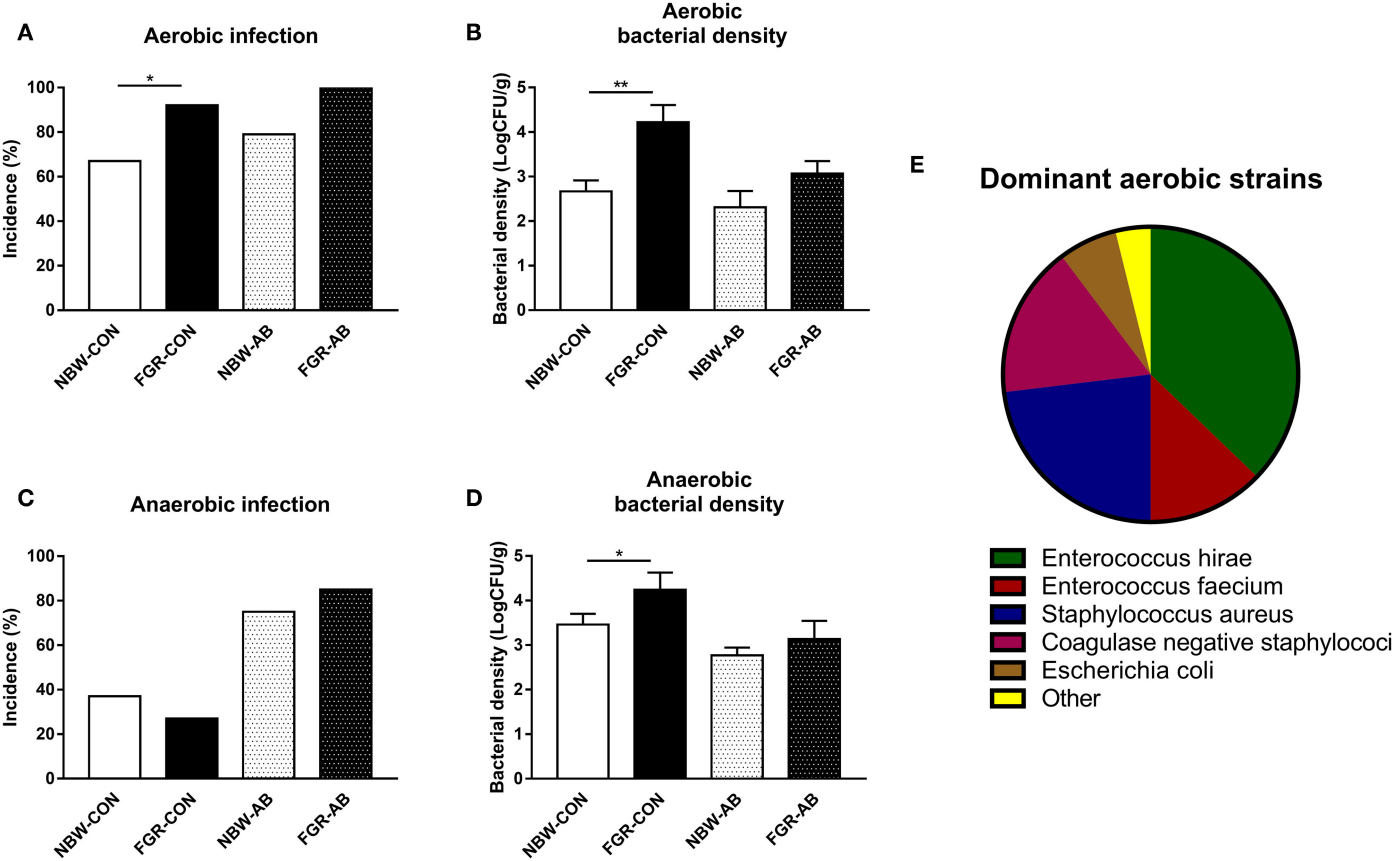
Spontaneous bacterial infection of the bone marrow of normal birth weight or fetal growth restricted preterm pigs, reared for 9 days, receiving either antibiotics (NBW-AB, *n* = 22 and FGR-AB, *n* = 8) or not (NBW-CON, *n* = 78 and FGR-CON, *n* = 19). **(A)** Incidence of aerobically cultured bacterial growth in the bone marrow. **(B)** Density of aerobically cultured bacteria in the bone marrow. **(C)** Incidence of anaerobically cultured bacterial growth in the bone marrow. **(D)** Density of anaerobically cultured bacteria in the bone marrow. **(E)** Distribution of dominant aerobically cultured bacterial strains. **(A,C)** Presented as fraction of animals with bacterial growth. **(B,D)** Presented as means with corresponding standard error. **(E)** Each bacterial group, shown as a fraction of the whole. *: Difference between either NBW-CON and FGR-CON or NBW-AB and FGR-AB; *P < 0.05, **P < 0.01.

In *Exp 3*, the overall birth weight were significantly lower in FGR than NBW animals (740 ± 30 vs. 1,141 ± 19 g, *P* < 0.001). At euthanasia on day 9, FGR-CON pigs showed a higher incidence of spontaneous aerobic bacterial infection of the bone marrow than their NBW-CON counterparts (*P* < 0.05, Figure 4A). The effect was still significant when comparing all FGR preterm pigs to all NBW (95 vs. 67%, Fisher's exact test: *P* < 0.01). The density of aerobic bacteria in the bone marrow was higher in FGR-CON pigs compared to NBW-CON (*P* < 0.01), but not in FGR-AB compared to NBW-AB (Figure 4B). When comparing all pigs, FGR preterm pigs had higher densities of aerobic bacteria in the bone marrow compared to NBW (3.9 vs. 2.6 × 10^9^ CFU/ml, *P* < 0.01). The incidence of anaerobic bacterial infection in bone marrow did not differ between groups (Figure 4C). However, the density of anaerobically cultured bacteria was higher in FGR-CON than NBW-CON (*P* < 0.05, Figure 4D). The dominant strains of aerobically cultured bacteria isolated from the bone marrow are shown in Figure 4E with the dominance of *Enterococcus* and *Staphyloccocus* spp.

**Figure 5:**
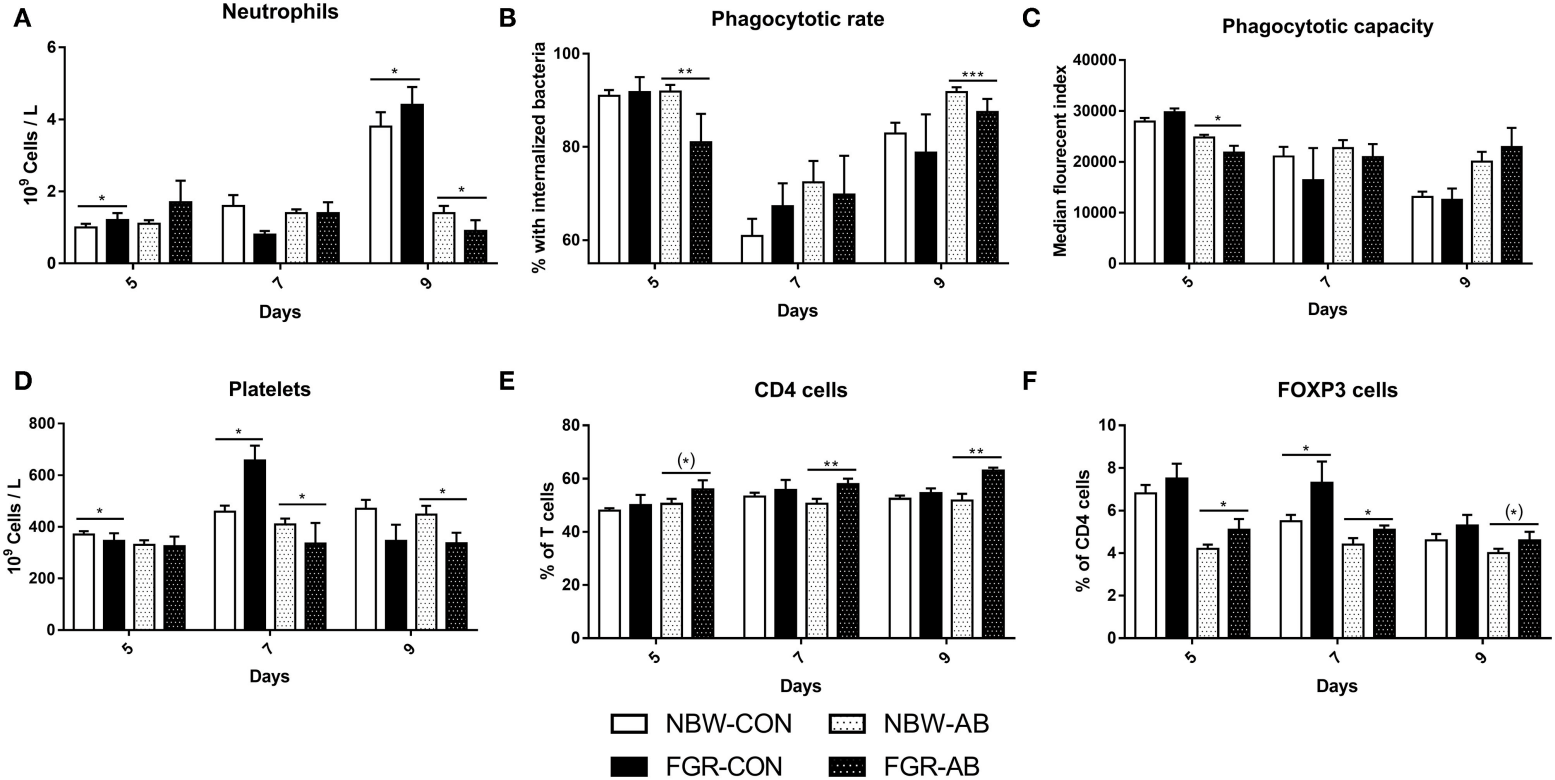
Blood immune cell subsets of normal birth weight or fetal growth restricted preterm pigs, reared for 9 days, receiving either antibiotics (NBW-AB, *n* = 13–19 and FGR-AB, *n* = 5–7) or not (NBW-CON, *n* = 47–54 and FGR-CON, *n* = 12–14). **(A)** Blood neutrophil counts. **(B)** Neutrophil phagocytic rate. **(C)** Neutrophil phagocytic capacity. **(D)** Blood platelets counts. **(E)** Fraction of CD4 positive T cells, as a percentage of all T cells. **(F)** Fraction of regulatory T cells, as a percentage of CD4 positive T cells. Data are presented as means with corresponding standard error, 5, 7, and 9 days after birth. *: Difference between either NBW-CON and FGR-CON or NBW-AB and FGR-AB; (*)*P* < 0.1, *P < 0.05, **P < 0.01, ***P < 0.001.

For hematological parameters, FGR-CON pigs showed higher blood neutrophil counts on day 5 than NBW-CON (*P* < 0.05, Figure 5A). On day 9, FGR-CON still had higher blood neutrophil counts than NBW-CON, but FGR-AB had lower neutrophil counts than NBW-AB (all *P* < 0.05, Figure 5A). Blood neutrophil phagocytosis function was also affected, as FGR-AB treated pigs had a lower phagocytic rate on both day 5 and 9 (*P* < 0.01 and 0.001, respectively), with a correspondingly lower phagocytic capacity only on day 5 (*P* < 0.05, Figures 5B,C). When comparing all FGR pigs to all NBW, only the phagocytic rate at day 5 differed, being lower in FGR pigs (85 ± 4 vs. 91 ± 1%, *P* < 0.01). Blood platelet counts were lower in FGR-CON than NBW-CON on day 5, but higher on day 7 (*P* < 0.05, Figure 5D). For FGR-AB, the platelets counts were lower on day 7 and 9 than in NBW-AB (all *P* < 0.05, Figure 5D). For blood T cell subsets in non-antibiotics treated animals, the fraction of regulatory T cells was higher on day 7 in the FGR-CON group relative to NBW-CON (*P* < 0.05). In antibiotics treated animals, FGR-AB had increased fractions of both CD4 positive and regulatory T cells on days 5, 7, and 9 compared to NBW-AB (*P* < 0.05, except CD4 positive T cells on day 5 and regulatory T cells on day 9, *P* < 0.01, Figures 5E,F). In addition, the ratio of CD4 to CD8 positive T cells was increased in FGR-AB pigs, compared to NBW-AB on day 9 (9.2 ± 1.1 vs. 5.8 ± 0.6, *P* < 0.001). Besides these findings, FGR pigs showed higher levels of total leucocytes, red blood cells, and hematocrit over the course of the experiment, compared to their NBW counterparts (Supplementary Table 2).

**Figure 6:**
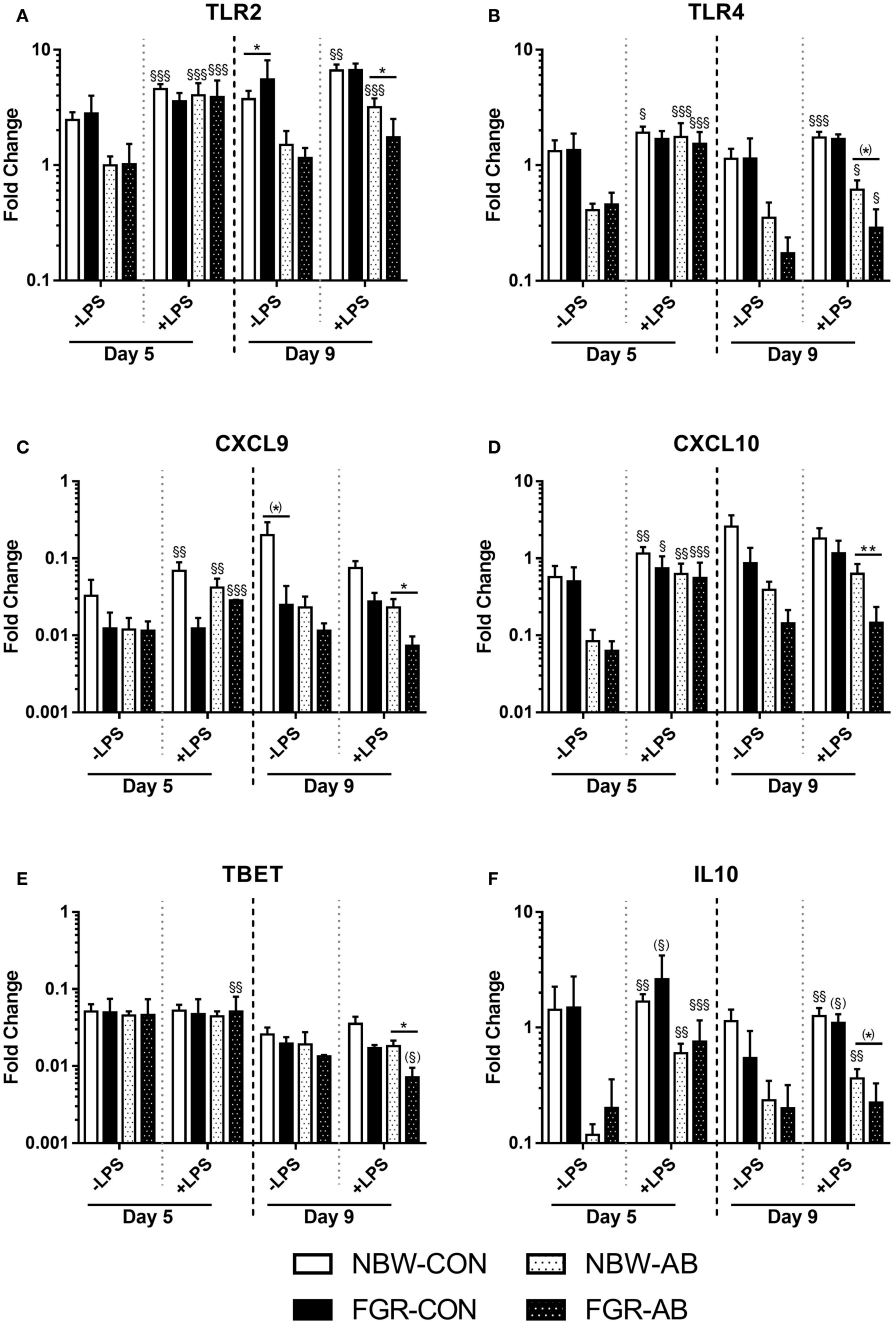
Blood leucocyte gene expressions of normal birth weight or fetal growth restricted preterm pigs, reared for 9 days, receiving either antibiotics (NBW-AB, *n* = 8–17 and FGR-AB, *n* = 2–5) or not (NBW-CON, *n* = 14–22 and FGR-CON, *n* = 3). **(A–F)** Expression of *TLR2, TLR4, CXCL9, CXCL10, TBET*, and *IL10*. Data are presented as means with corresponding standard error, 5 and 9 days after birth. *: Difference between either NBW-CON and FGR-CON or NBW-AB and FGR-AB; (*): *P* < 0.1, *P < 0.05, **P < 0.01. ^§^: Effect of LPS stimulation; (^§^):P < 0.1, ^§^*P* < 0.05, ^§§^*P* < 0.01, ^§§§^*P* < 0.001.

Gene expression analysis after blood stimulation on day 5 and 9 was performed to support the hematological and T cell findings (Figures 6A–F). On day 5, baseline gene expressions did not differ between FGR-CON and NBW-CON or FGR-AB or NBW-AB. After stimulation with LPS, expressions of *TLR2, TLR4*, and *CXCL9* were increased only in NBW-CON pigs, but not FGR-CON (all *P* < 0.05, Figures 6A–C). For these same genes, LPS effects were similar in FGR-AB and NBW-AB pigs. Only *TBET* was expressed slightly more in FGR-AB pigs after LPS stimulation, than in NBW-AB (*P* < 0.01, Figure 6E). By day 9, the baseline expression of *TLR2* was higher in FGR-CON pigs than NBW-CON (*P* < 0.05, Figure 6A) but tended to be lower for *CXCL9* (*P* = 0.07, Figure 6C). Furthermore, FGR-CON pigs were not able to mount expressions of *TLR2* and *TLR4* after stimulation with LPS, whereas NBW-CON pigs were more competent (*P* < 0.01 and 0.001, respectively, Figures 6A,B). Baseline expression of genes did not differ between FGR-AB and NBW-AB on day 9. However, after stimulation with LPS, FGR-AB pigs showed lower expression of *TLR2, CXCL9, CXCL10*, and *TBET* than NBW-AB (all *P* < 0.05, Figures 1A,C–E) with a tendency toward lower expression of *TLR4* and *IL10* (*P* = 0.07 and 0.06, respectively, Figures 6B,F). Likewise, the FGR-AB pigs did not increase expression of *IL10* after LPS stimulation, whereas NBW-AB did (*P* < 0.01, Figure 6F). For the remaining tested genes, there were no or minor differences among the groups (Supplementary Table 3).

## Discussion

Following preterm birth, immune status, and infection resistance during the first days and weeks of life are critical factors for survival and long-term health. We show that preterm pigs born following fetal growth-restriction, show clear evidence of impaired immune competence in the critical neonatal period, with adverse clinical outcome after infection challenge and an increased rate of spontaneous infections. Interestingly, the effects of being born growth restricted were in part exacerbated by antibiotic use in the immediate postnatal period, although more studies are required to confirm this and investigate mechanisms. Collectively, our data help to explain why infants born both premature and SGA have increased morbidity and mortality. Although antibiotics seemed to reduce bacterial densities in bone marrow, the results suggest a need for increased critical care, but also cautious antibiotics use, for this special subpopulation of preterm newborn infants.

Cord blood of FGR and NBW pigs was initially used to examine immune competence at birth by a whole blood stimulation assay with LPS. Before LPS stimulation, FGR preterm pigs showed lower baseline expressions of few genes, However, we did not measure the leucocyte levels in *Exp 1*, but we have previously shown that FGR piglets tend to have lower lymphocyte counts (but not neutrophil and monocyte counts) in cord blood (10). Therefore, it is possible that differences in expressions of some lymphocytes-related genes at baseline may be influenced by the leucocyte levels. Still, after stimulation with LPS, it appeared that blood leucocytes of FGR animals had a diminished response with regards to several genes related to innate and adaptive immunity as well as cellular metabolism. This indicates an overall impaired capacity to mount sufficient immune response to infectious challenges in newborn preterm pigs with fetal growth restriction. This is similar to the diminished immune function at birth observed in cord blood of SGA preterm infants (17).

To be able to relate the *ex vivo* immune response to the actual infection outcomes in FGR preterm pigs, we performed an *in vivo* infection challenge with systemic administration of SE to induce neonatal sepsis (*Exp 2*), in a similar manner to previous studies (29). SE is normally considered a low virulent pathogen and was previously often considered a contamination if cultured from the blood (33). However, coagulase negative staphylococci, like SE, are among the most common organism isolated from preterm infants with LOS (3, 34). As expected, SE infected pigs appeared to undergo respiratory acidosis 6 h after infusion (low blood pH and oxygen pressure with high carbon dioxide pressure), similar to what have been observed in preterm infants with neonatal sepsis (35). The overall effects of SE challenge, when compared to saline infused pigs (drop in pH and leucocyte subsets after SE inoculation), were also similar to those that we have observed in previous experiments, using the same model (29). This confirms that pigs underwent a relevant immunological challenge. Interestingly, SE infected FGR animals showed more severe clinical and cellular responses, relative to their littermates of adequate birth weight. Their drop in blood pH and bicarbonate was followed by a corresponding rise in lactate, indicating that the FGR preterm pigs were undergoing a more severe septic response to the SE challenge. This was also accompanied by a more severe drop in neutrophils and platelets in SE-FGR pigs. Collectively, it was clear that newborn growth restricted preterm pigs were more affected by the SE inoculation than their preterm normal birth weight counterparts. The combined data in *Exp 1* and *2* suggest that infants born with prematurity and fetal growth restriction have impaired immune competence that may lead to higher susceptibility to neonatal sepsis following systemic infection, in the immediate neonatal period.

We also attempted to characterize the immune development and test the susceptibility to spontaneous infection, over the first few days after preterm birth in FGR pigs. It was evident that FGR preterm pigs, were more prone to infection of aerobically cultured bacteria in their bone marrow, with higher densities of bacteria than pigs of adequate birth weight. The effect was less apparent for antibiotics treated pigs that showed bacterial densities comparable to the NBW-CON pigs. For anaerobically cultured bacteria there were no effects, possibly a result of the high blood flow in the bone marrow, somewhat restricting anaerobic bacterial growth. In fact, very few strictly anaerobic bacteria could be cultured (data not shown). The species of aerobically cultured bacteria found in the bone marrow are all known to inhabit the gut of preterm infants and pigs (34, 36). This indicates that preterm SGA infants may be either more prone to bacterial translocation across the gut or have a reduced capacity to clear the gut derived systemic bacteria. In this study, we have not investigated gut permeability *per se*, so differentiating between the two factors is difficult. The supporting data showed that over the first week of life, FGR preterm pigs consistently had poorer systemic immune functions and an immune suppressed status, with less capacity to response to an *ex vivo* challenge.

The development of immune cell subsets, including T cell subsets, was affected by neonatal antibiotic treatment, as expected (24). Interestingly though, adverse immune effects of being born FGR were more pronounced in animals treated with antibiotics. The FGR-AB preterm pigs showed lower neutrophil counts with poorer phagocytosis capacity, as well as higher fractions of CD4 positive and regulatory T cells, relative to NBW-AB. The FGR-CON pigs also showed higher counts of regulatory T cells on day 7, indicating the effect of low birth weight was not entirely dependent on antibiotics. These results suggest that FGR preterm pigs, irrespective of antibiotic treatment, had decreased immune function, relative to their NBW counterparts. The whole blood LPS stimulation assays could support this conclusion in that blood leucocytes of FGR-AB pigs expressed less *TBET* than NBW-AB pigs, indicating that the increased number of CD4 positive T cells in FGR-AB pigs were skewed toward a Th2 phenotype. As in *Exp 1*, the baseline leucocyte gene expression levels could be influenced by the differences in neutrophil counts observed. However, several innate immune related genes were also expressed less in FGR-AB and FGR-CON pigs following LPS stimulation than in their NBW counterparts. The increased fraction of regulatory T cells may act to dampen the immune competence or delay immune maturation in FGR preterm pigs. This immune suppressive status may lead to less systemic bacterial clearance and more bacterial accumulation in the bone marrow of FGR preterm pigs. However, these longer term immune effects are more suggestive and require further exploration.

Interestingly, in *Exp 1*, we found that cord blood leucocytes of newborn growth restricted preterm pigs had a lower expression of genes related to cellular energy metabolism (*PPARA, HIF1A*, and *PKM*), both before and after LPS stimulation. The *PPARA* gene encodes the protein peroxisome proliferator–activated receptor α, which is crucial for fatty acid metabolism and ketogenesis (37). The gene is mostly expressed in hepatocytes, where it plays a major role in fasting responses (38). However, higher levels of peroxisome proliferator–activated receptors in monocytes has been linked to anti-inflammatory cytokine production and differentiation to macrophages (39–41) as well as increased activity of cellular fatty acid oxidation and oxidative phosphorylation (42). Pyruvate kinase (encoded by *PKM*) is a glycolytic enzyme crucial for the generation of adenosine triphosphate (ATP), the last step of the glycolysis process, normally occurring under anaerobic conditions (43). However, it is well-established that pro-inflammatory leucocytes rely on glycolysis to exude their function, even when oxygen is abundant, a phenomenon known as the Warburg effect (44, 45). Hypoxia-inducible factor-1a (encoded by *HIF1A*) is also a regulator in glycolysis and has been proposed as a mediator of the Warburg effect (46, 47) via the mTOR immune pathway and therefore can also be considered an immune related factor (48, 49). The drop in expressions of *PKM, HIF1A*, and *PPARA* in *Exp 1* may reflect less ATP production, possibly due to lower energy reservoirs, and therefore could impair energy-consuming immune responses in the FGR preterm pigs. This observation was similar to the poor immune responses and low expressions of monocyte genes related to both glycolysis and oxidative phosphorylation in preterm vs. term infants (50). Further, following SE inoculation in *Exp 2*, the FGR pigs had higher glucose levels, which could indicate a dysregulated metabolism during infections or endogenous glucose generation associated with more excessive inflammatory response. This could possibly be part of the explanation for their poorer outcome, compared to NBW preterm piglets subjected to the same experimental SE inoculation.

Preterm pigs have emerged as good models for preterm infants, with many similarities in size, physiology, and immune system (27, 32). Due to the large litter sizes of pigs there is a sizable variation in birth weight, making it possible to compare low and high birth weight individuals (51). The causes of this birth weight variation may be a combination of placental variation in blood flow and intrinsic fetal genetic determinants. In humans, the causes leading to slow intrauterine growth are more diverse, including other associated pathologies, like maternal infections and preeclampsia playing a role (52). These conditions could separately affect postnatal immune development, regardless of birth weight. Using elective cesarean section of preterm pigs from uncomplicated sow pregnancies, we can study the effects of fetal growth restriction, independent of such inflammatory and pathological maternal conditions, leading to preterm birth with/without SGA in infants. Since there were no prenatal complications prior to delivery of piglets, the observed immunity effects in this study are likely to arise from slow intrauterine growth *per se*, independent of any fetal or maternal inflammatory or pathological conditions.

We conclude that there are clear effects on the neonatal immune system of being born SGA after preterm birth, with greater sensitivity and adverse response to bacterial infection, less responsive leucocytes *in vitro*, and increased fraction of regulatory T cells. Collectively, our results suggest that preterm pigs born moderately growth restricted were immune suppressed or experienced some delay in immune maturation, which may be further exacerbated by neonatal antibiotic use. Such effects may be most pronounced in the immediate neonatal period (e.g., the first 1–2 weeks), as supported by the limited effects of moderate FGR and prematurity on blood immune parameters (10, 53) and gut and brain development beyond the first 2 weeks of life (53, 54). Correspondingly, a dysfunctional immune system and an increased risk of infections in preterm infants with fetal growth restriction suggest special care and medical attention in this population to avoid damaging infections in the critical neonatal period.

## Data Availability Statement

The raw data supporting the conclusions of this article will be made available by the authors, without undue reservation.

## Ethics Statement

The animal study was reviewed and approved by Danish National Committee on Animal Experimentation (2014-15-0201-00418).

## Author Contributions

DN designed the study. DN, AB, and SR carried out the experiments and laboratory analysis. OB performed data analysis and wrote the manuscript. OB, SR, AB, PS, and DN provided critical interpretation and revised the manuscript. OB and DN had primary responsibility for the final content. All authors approved the final paper.

## Conflict of Interest

The authors declare that the research was conducted in the absence of any commercial or financial relationships that could be construed as a potential conflict of interest.
