# Supplementary material for: Impaired Neonatal Immunity and Infection Resistance Following Fetal Growth Restriction in Preterm Pigs
Source: Front Immunol. 2020 Aug 13;11:1808. doi: 10.3389/fimmu.2020.01808 (PMC7438575; doi:10.3389/fimmu.2020.01808)
Supplement: Supplementary file 1 [file Table_1.docx]

**Supplementary tables**

**Supplementary table 1: Hematological parameters in *Exp 2***

|  | **Time after**  **SE (hours)** | **CON**  **(n = 19)** | **SE-NBW**  **(n = 29)** | **SE-FGR**  **(n = 9)** | **P**  **SE** | **P**  **FGR** |
| --- | --- | --- | --- | --- | --- | --- |
| *Total leucocytes (10^9^ cells/L)* | *6* | 3.1 (0.2) | 1.4 (0.1) | 1.8 (0.3) | NS | NS |
|  | *12* | 3.1 (0.2) | 1.7 (0.2) | 2.4 (0.6) | NS | NS |
|  | *24* | 2.3 (0.2) | 1.9 (0.2) | 1.9 (0.4) | NS | NS |
| *Neutrophils (10^9^ cells/L)* | *6* | 1.0 (0.1) | 0.6 (0.1) | 0.6 (0.1) | < 0.001 | NS |
|  | *12* | 1.1 (0.1) | 0.9 (0.1) | 0.5 (0.2) | < 0.01 | < 0.05 |
|  | *24* | 0.8 (0.1) | 1.0 (0.2) | 1.1 (0.4) | NS | NS |
| *Lymphocytes (10^9^ cells/L)* | *6* | 2.0 (0.2) | 0.8 (0.1) | 1.1 (0.1) | < 0.001 | < 0.001 |
|  | *12* | 1.9 (0.1) | 0.7 (0.1) | 1.8 (0.5) | < 0.001 | < 0.01 |
|  | *24* | 1.4 (0.1) | 0.8 (0.1) | 0.7 (0.1) | < 0.001 | NS |
| *Monocytes (10^9^ cells/L)* | *6* | 0.08 (0.01) | 0.02 (0.00) | 0.06 (0.03) | < 0.001 | < 0.05 |
|  | *12* | 0.08 (0.01) | 0.03 (0.00) | 0.05 (0.01) | < 0.001 | < 0.05 |
|  | *24* | 0.05 (0.01) | 0.04 (0.01) | 0.09 (0.05) | < 0.05 | NS |
| *Platelets (10^9^cells/L)* | *6* | 336 (6) | 289 (11) | 271 (13) | NS | NS |
|  | *12* | 312 (10) | 256 (12) | 193 (25) | < 0.001 | < 0.001 |
|  | *24* | 243 (19) | 186 (11) | 164 (11) | < 0.001 | NS |
| *Red blood cells (10^12^cells/L)* | *6* | 4.5 (0.1) | 4.2 (0.1) | 4.3 (0.2) | NS | NS |
|  | *12* | 4.2 (0.1) | 4.0 (0.1) | 3.6 (0.3) | NS | NS |
|  | *24* | 4.2 (0.1) | 3.9 (0.1) | 3.7 (0.2) | NS | NS |
| *Hemoglobin (g/L)* | *6* | 6.1 (0.1) | 5.9 (0.2) | 6.1 (0.1) | NS | NS |
|  | *12* | 5.5 (0.1) | 5.6 (0.1) | 5.0 (0.4) | 0.09 | 0.08 |
|  | *24* | 5.3 (0.1) | 5.2 (0.1) | 5.0 (0.1) | NS | NS |
| *Hematocrit (%)* | *6* | 33 (1) | 32 (1) | 34 (1) | NS | NS |
|  | *12* | 31 (1) | 31 (1) | 28 (2) | NS | NS |
|  | *24* | 30 (1) | 28 (1) | 28 (1) | NS | NS |

Hematological parameters for preterm animals 6, 12 and 24 hours after infusion with *Staphylococcus epidermidis* (SE) or control saline. SE infused preterm pigs of normal birth weight (SE-NBW) and of low birth weight (SE-FGR) are compared; likewise all saline infused preterm pigs (CON) are compared to all SE infused. Data presented as means with corresponding standard error, P values less than 0.1 are presented and P values less than 0.05 are considered significant. NS: Not significant.

**Supplementary table 2: Hematological parameters in *Exp 3.***

|  | **Day** | **NBW-CON**  **(n = 47-54)** | **FGR-CON**  **(n = 12-14)** | **P** | **NBW-AB**  **(n = 13-19)** | **FGR-AB**  **(n = 5-7)** | **P** |
| --- | --- | --- | --- | --- | --- | --- | --- |
| *Total leucocytes (10^9^ cells/L)* | *5* | 2.2 (0.1) | 2.4 (0.3) | < 0.01 | 2.8 (0.1) | 3.5 (0.6) | 0.07 |
|  | *7* | 3.5 (0.4) | 2.8 (0.4) | NS | 3.5 (0.2) | 3.0 (0.4) | 0.1 |
|  | *9* | 5.9 (0.5) | 6.2 (0.6) | 0.06 | 2.9 (0.3) | 2.4 (0.8) | NS |
| *Lymphocytes (10^9^ cells/L)* | *5* | 1.0 (0.0) | 1.1 (0.1) | NS | 1.6 (0.1) | 1.7 (0.2) | NS |
|  | *7* | 1.6 (0.1) | 1.8 (0.3) | NS | 1.9 (0.1) | 1.4 (0.2) | < 0.001 |
|  | *9* | 1.7 (0.1) | 1.4 (0.2) | NS | 1.4 (0.1) | 1.4 (0.4) | NS |
| *Monocytes (10^9^ cells/L)* | *5* | 0.09 (0.01) | 0.12 (0.03) | NS | 0.04 (0) | 0.04 (0.01) | NS |
|  | *7* | 0.16 (0.03) | 0.15 (0.06) | NS | 0.08 (0.02) | 0.05 (0.01) | NS |
|  | *9* | 0.19 (0.02) | 0.18 (0.06) | NS | 0.06 (0.01) | 0.07 (0.04) | NS |
| *Red blood cells (10^12^cells/L)* | *5* | 3.7 (0.1) | 4.0 (0.1) | < 0.05 | 3.8 (0.1) | 4.2 (0.3) | NS |
|  | *7* | 3.7 (0.1) | 3.9 (0.5) | NS | 3.7 (0.1) | 3.4 (0.2) | NS |
|  | *9* | 3.5 (0.2) | 3.8 (0.1) | < 0.05 | 3.5 (0.1) | 3.6 (0.2) | NS |
| *Hemoglobin (g/L)* | *5* | 4.8 (0.1) | 5.2 (0.1) | < 0.01 | 5.0 (0.1) | 5.4 (0.4) | NS |
|  | *7* | 4.5 (0.1) | 4.9 (0.6) | NS | 4.6 (0.2) | 4.4 (0.3) | NS |
|  | *9* | 4.1 (0.1) | 4.6 (0.2) | < 0.05 | 4.2 (0.1) | 4.3 (0.3) | NS |
| *Hematocrit (%)* | *5* | 26.8 (0.5) | 29.5 (0.7) | < 0.01 | 28.3 (0.6) | 31.0 (2.5) | NS |
|  | *7* | 25.4 (0.3) | 27.0 (3.2) | NS | 26.4 (0.9) | 25.0 (1.7) | NS |
|  | *9* | 23.3 (0.5) | 25.7 (1.0) | < 0.05 | 23.8 (0.7) | 24.1 (1.9) | NS |
| *T cells (% of lymphocytes)* | 5 | 61.6 (1.5) | 60.1 (2.9) | NS | 64.7 (1.5) | 61.4 (3.7) | NS |
|  | 7 | 65.8 (1.1) | 66.4 (2.8) | NS | 65.2 (1.2) | 63.5 (2.3) | NS |
|  | 9 | 59.3 (1.5) | 52.4 (2.8) | NS | 66.4 (2.1) | 66.3 (2.9) | NS |
| *CD4+ T cells (% of T cells)* | 5 | 47.9 (1.0) | 50.1 (1.3) | NS | 50.5 (1.9) | 55.9 (3.5) | 0.07 |
|  | 7 | 53.3 (1.4) | 55.7 (3.8) | NS | 50.6 (1.8) | 57.9 (2.1) | < 0.01 |
|  | 9 | 52.4 (1.2) | 54.5 (1.8) | NS | 51.8 (2.5) | 63.0 (1.1) | < 0.01 |
| *CD8+ T cells (% of T cells)* | 5 | 8.5 (0.6) | 9.3 (1.1) | NS | 8.5 (1.1) | 9.2 (1.5) | NS |
|  | 7 | 9.3 (0.5) | 7.6 (1.2) | NS | 10.7 (0.6) | 9.8 (1.1) | NS |
|  | 9 | 10.7 (0.6) | 10.8 (1.0) | NS | 9.5 (0.7) | 7.7 (0.7) | 0.1 |
| *FOXP3+ T cells (% of CD+ T cells)* | 5 | 6.8 (0.4) | 7.5 (0.7) | NS | 4.2 (0.2) | 5.1 (0.5) | < 0.05 |
|  | 7 | 5.5 (0.3) | 7.3 (1.0) | < 0.05 | 4.4 (0.3) | 5.1 (0.2) | < 0.05 |
|  | 9 | 4.6 (0.3) | 5.3 (0.5) | NS | 4.0 (0.2) | 4.6 (0.4) | 0.09 |

Hematological parameters for preterm pigs 5, 7 and 9 days after birth. Animals of normal and low birth weight, not receiving antibiotics (NBW-CON and FGR-CON) were compared; likewise normal and low birth weight animals receiving antibiotics (NBW-AB and FGR-AB) were compared. Data presented as means with corresponding standard error, P values less than 0.1 are presented and P values less than 0.05 are considered significant. NS: Not significant.

**Supplemental table 3: Gene expression analysis in *Exp 3*.**

| **Gene** | **Day** | **Stimulation** | **NBW-CON** | **FGR-CON** | **P** | **NBW-AB** | **FGR-AB** | **P** |
| --- | --- | --- | --- | --- | --- | --- | --- | --- |
| *GATA 3* | 5 | -LPS | 0.07 (0.01) | 0.11 (0.01) | < 0.05 | 0.16 (0.03) | 0.12 (0.04) | NS |
|  |  | +LPS | 0.08 (0.01) | 0.13 (0.02) | < 0.05 | 0.16 (0.04) | 0.13 (0.03) | NS |
|  | 9 | -LPS | 0.05 (0.01) | 0.04 (0.01) | NS | 0.07 (0.02) | 0.05 (0.02) | NS |
|  |  | +LPS | 0.05 (0.01) | 0.04 (0.01) | NS | 0.05 (0.01) | 0.07 (0.03) | NS |
| *TNFA* | 5 | -LPS | 3.51 (1.88) | 1.35 (1.05) | NS | 0.44 (0.09) | 0.76 (0.33) | NS |
|  |  | +LPS | 2.76 (0.79)^§^ | 2.06 (1.11)^§§^ | NS | 0.65 (0.12)^§§^ | 1.43 (0.82)^§§^ | NS |
|  | 9 | -LPS | 1.96 (0.47) | 1.45 (0.69) | NS | 1.30 (0.60) | 1.20 (0.75) | NS |
|  |  | +LPS | 1.64 (0.38) | 1.45 (0.34) | NS | 1.04 (0.34) | 0.87 (0.65)^§^ | NS |
| *IL2* | 5 | -LPS | 0.11 (0.07) | 0.01 (0.00) | NS | 0.05 (0.02) | 0.04 (0.01) | NS |
|  |  | +LPS | 0.05 (0.04) | 0.01 (0.00) | NS | 0.05 (0.02) | 0.02 (0.01) | NS |
|  | 9 | -LPS | 0.09 (0.04) | 0.01 (0.01) | NS | 0.08 (0.04) | 0.06 (0.05) | NS |
|  |  | +LPS | 0.12 (0.06) | 0.11 (0.10)^§§^ | NS | 0.03 (0.03) | 0.03 (0.03)^§^ | NS |
| *IL4* | 5 | -LPS | 0.24 (0.10) | 0.14 (0.02) | NS | 0.33 (0.11) | 0.32 (0.05) | NS |
|  |  | +LPS | 0.28 (0.09) | 0.13 (0.04) | NS | 0.31 (0.08) | 0.39 (0.09) | NS |
|  | 9 | -LPS | 0.04 (0.01) | 0.03 (0.01) | NS | 0.06 (0.01) | 0.06 (0.02) | NS |
|  |  | +LPS | 0.05 (0.01) | 0.03 (0.01) | NS | 0.05 (0.02) | 0.06 (0.02) | < 0.05 |
| *IL6* | 5 | -LPS | 4.15 (2.71) | 5.29 (5.14) | NS | 0.13 (0.02) | 0.16 (0.04) | NS |
|  |  | +LPS | 2.06 (1.24)^§^ | 7.57 (6.81)^(§)^ | NS | 0.31 (0.07)^§§^ | 0.26 (0.09)^§§§^ | NS |
|  | 9 | -LPS | 1.05 (0.29) | 1.75 (0.87) | NS | 0.62 (0.33) | 0.97 (0.74) | NS |
|  |  | +LPS | 1.07 (0.26) | 0.75 (0.50) | NS | 0.76 (0.31) | 0.88 (0.63) | NS |
| *IL12* | 5 | -LPS | 0.18 (0.07) | 0.04 (0.01) | NS | 0.09 (0.03) | 0.38 (0.17) | < 0.05 |
|  |  | +LPS | 0.20 (0.12) | 0.02 (0.00)^§^ | NS | 0.17 (0.05)^§§^ | 0.70 (0.67) | NS |
|  | 9 | -LPS | 0.28 (0.08) | 0.10 (0.04) | NS | 0.10 (0.04) | 0.13 (0.08) | NS |
|  |  | +LPS | 0.35 (0.09) | 0.48 (0.23) | NS | 0.10 (0.04) | 0.09 (0.07)^§^ | NS |
| *IL17* | 5 | -LPS | 0.28 (0.12) | 0.06 (0.04) | NS | 0.09 (0.04) | 0.16 (0.09) | NS |
|  |  | +LPS | 0.23 (0.13) | 0.07 (0.06) | NS | 0.14 (0.07) | 0.84 (0.82) | NS |
|  | 9 | -LPS | 0.45 (0.14) | 0.11 (0.07) | NS | 0.12 (0.06) | 0.09 (0.05) | NS |
|  |  | +LPS | 0.55 (0.14) | 0.43 (0.21)^§^ | NS | 0.10 (0.06) | 0.10 (0.08) | NS |
| *RORA* | 5 | -LPS | 0.04 (0.01) | 0.03 (0.00) | NS | 0.02 (0.00) | 0.04 (0.02) | NS |
|  |  | +LPS | 0.06 (0.02) | 0.03 (0.00) | NS | 0.02 (0.01) | 0.02 (0.01)^§^ | NS |
|  | 9 | -LPS | 0.02 (0.01) | 0.04 (0.01) | NS | 0.01 (0.00) | 0.01 (0.00) | < 0.05 |
|  |  | +LPS | 0.03 (0.01) | 0.03 (0.01) | NS | 0.01 (0.00) | 0.01 (0.00) | NS |
| *S100A9* | 5 | -LPS | 16.0 (4.13) | 19.7 (14.1) | NS | 1.25 (0.38) | 1.02 (0.48) | NS |
|  |  | +LPS | 25.2 (5.25)^§§^ | 22.8 (16.8)^§§§^ | NS | 2.50 (0.51)^§§§^ | 1.47 (1.04) | NS |
|  | 9 | -LPS | 10.5 (2.81) | 16.0 (7.36) | NS | 12.4 (7.09) | 7.29 (5.54) | NS |
|  |  | +LPS | 15.6 (3.30)^§§§^ | 20.8 (10.5) | NS | 11.7 (4.32)^§§^ | 5.17 (3.06) | NS |
| *MPO* | 5 | -LPS | 0.11 (0.06) | 0.03 (0.01) | NS | 0.05 (0.02) | 0.04 (0.02) | NS |
|  |  | +LPS | 0.09 (0.04) | 0.02 (0.01)^§§§^ | NS | 0.06 (0.02) | 0.04 (0.01)^§^ | NS |
|  | 9 | -LPS | 0.13 (0.05) | 0.02 (0.01) | NS | 0.07 (0.04) | 0.05 (0.05) | NS |
|  |  | +LPS | 0.14 (0.06) | 0.13 (0.11) | NS | 0.05 (0.03) | 0.04 (0.04) | NS |
| *HIF1A* | 5 | -LPS | 1.47 (0.20) | 1.14 (0.27) | NS | 2.00 (0.29) | 1.53 (0.46) | NS |
|  |  | +LPS | 2.36 (0.45)^§^ | 1.51 (0.48)^(§)^ | NS | 2.22 (0.31)^§^ | 1.66 (0.63) | NS |
|  | 9 | -LPS | 1.55 (0.21) | 1.82 (0.74) | NS | 1.02 (0.22) | 1.08 (0.50) | NS |
|  |  | +LPS | 1.52 (0.17) | 1.36 (0.44) | NS | 0.99 (0.15) | 0.90 (0.30)^(§)^ | NS |
| *PPARA* | 5 | -LPS | 0.09 (0.01) | 0.06 (0.02) | < 0.05 | 0.08 (0.01) | 0.11 (0.00) | < 0.05 |
|  |  | +LPS | 0.14 (0.03) | 0.05 (0.01) | < 0.05 | 0.12 (0.02)^§^ | 0.13 (0.01) | NS |
|  | 9 | -LPS | 0.03 (0.00) | 0.03 (0.01) | NS | 0.03 (0.00) | 0.05 (0.01) | NS |
|  |  | +LPS | 0.03 (0.00) | 0.03 (0.01) | NS | 0.03 (0.00) | 0.04 (0.00) | < 0.001 |
| *PADHA1* | 5 | -LPS | 0.60 (0.07) | 0.50 (0.05) | NS | 0.63 (0.07) | 0.74 (0.14) | NS |
|  |  | +LPS | 0.48 (0.05)^§^ | 0.42 (0.03)^(§)^ | NS | 0.77 (0.11) | 1.18 (0.41) | NS |
|  | 9 | -LPS | 0.37 (0.04) | 0.41 (0.07) | NS | 0.33 (0.05) | 0.26 (0.06) | NS |
|  |  | +LPS | 0.42 (0.05) | 0.72 (0.17) ^(§)^ | < 0.05 | 0.29 (0.03) | 0.28 (0.10) | NS |
| *PKM* | 5 | -LPS | 6.25 (0.57) | 5.35 (0.42) | NS | 5.06 (0.63) | 6.20 (2.91) | NS |
|  |  | +LPS | 6.35 (1.03) | 4.14 (0.82)^(§)^ | NS | 4.57 (0.57) | 3.89 (1.55)^(§)^ | NS |
|  | 9 | -LPS | 3.27 (0.55) | 5.36 (1.92) | NS | 2.97 (0.69) | 2.26 (0.82) | NS |
|  |  | +LPS | 4.29 (0.54)^§§^ | 5.02 (1.62) | NS | 2.22 (0.29) | 2.24 (0.65) | NS |
| *TGFB* | 5 | -LPS | 5.95 (1.67) | 4.68 (1.84) | NS | 8.32 (2.07) | 14.1 (4.64) | NS |
|  |  | +LPS | 7.29 (1.72)^§^ | 6.31 (2.55)^§§^ | NS | 11.7 (3.08) | 15.1 (2.71) | NS |
|  | 9 | -LPS | 2.13 (0.23) | 1.35 (0.22) | NS | 1.76 (0.32) | 1.67 (0.43) | NS |
|  |  | +LPS | 2.16 (0.28) | 1.89 (0.29) | NS | 1.37 (0.15) | 1.59 (0.35)^(§)^ | NS |
| *HK1* | 5 | -LPS | 0.24 (0.04) | 0.24 (0.03) | NS | 0.21 (0.03) | 0.18 (0.11) | 0.09 |
|  |  | +LPS | 0.37 (0.09)^§§^ | 0.28 (0.06) | NS | 0.24 (0.04) | 0.21 (0.11) | NS |
|  | 9 | -LPS | 0.24 (0.05) | 0.26 (0.09) | NS | 0.13 (0.04) | 0.17 (0.10) | NS |
|  |  | +LPS | 0.40 (0.06)^§§§^ | 0.32 (0.06)^(§)^ | NS | 0.15 (0.02)^§§^ | 0.19 (0.10)^§^ | NS |

Gene expressions for preterm pigs 5 and 9 days after birth. Animals of normal and low birth weight, not receiving antibiotics (NBW-CON and FGR-CON) were compared; likewise, normal and low birth weight animals receiving antibiotics (NBW-AB and FGR-AB) were compared. Gene expressions are shown as mean fold changes with corresponding standard error, before and after stimulation with lipopolysaccharide (LPS). Effect of LPS stimulation within a specific group; (§): P < 0.1, §: P < 0.05, §§ P < 0.01 §§§: P < 0.001. NS: Not significant.
